# P-428. Changes in Antibiotic Timing and Selection for Pediatric Acute Osteomyelitis Following the 2021 IDSA Guidelines

**DOI:** 10.1093/ofid/ofaf695.644

**Published:** 2026-01-11

**Authors:** Andrew Edgington, Deepa Mukundan, Mounika Polavarapu, Shipra Singh

**Affiliations:** University of Toledo College of Medicine and Life Sciences, Toledo, OH; University of Toledo College of Medicine and Life Sciences, Toledo, OH; University of Toledo College of Medicine and Life Sciences, Toledo, OH; University of Toledo College of Medicine and Life Sciences, Toledo, OH

## Abstract

**Background:**

In 2021, the IDSA updated its guidelines to support earlier intravenous (IV)-to-oral antibiotic transitions in children with uncomplicated acute hematogenous osteomyelitis (AHO). We examined a large real-world dataset to assess the impact of this change on IV therapy duration, oral step-down practices, and outcomes.

**Methods:**

Using a multi-center EHR network (TriNetX), we performed a retrospective cohort study of pediatric patients (0–17 years) with AHO who received IV antibiotics within 3 days of diagnosis. Two cohorts, pre-guideline (2018–20) and post-guideline (2022–24), were compared, including a subgroup analysis of those who transitioned to oral antibiotics within 14 days of IV initiation. The cohorts were propensity-matched 1:1 by age, sex, and race/ethnicity. We compared antibiotic use (IV vs oral) as well as complication, readmission, and surgical intervention rates, with outcomes tracked for 90 days.

**Results:**

Among 106 matched patients (53 per group), the median time to oral step-down was similar between pre- and post-guideline groups (3.5 vs 3.0 days). There was no significant change in IV antibiotic exposures per patient; however, piperacillin-tazobactam use increased from 0% to 18.8% after the update (p=0.0009). Complications (sepsis or requiring surgery) were lower in the post-guideline cohort (32.1% vs 49.1%), but this difference was not significant (p=0.075). No deaths occurred in either group.

**Conclusion:**

Although the overall duration of IV therapy did not decrease significantly, earlier oral transitions and prescribing changes indicate partial adoption of the 2021 IDSA guidelines. Modest changes in antibiotic selection and a trend toward fewer complications post-guideline suggest gradual adoption of updated recommendations. Further study is needed to assess long-term outcomes and institutional practice variation.

**Disclosures:**

All Authors: No reported disclosures

